# The twin pillars of Disease Models & Mechanisms

**DOI:** 10.1242/dmm.048951

**Published:** 2021-02-22

**Authors:** E. Elizabeth Patton,

**Affiliations:** MRC Human Genetics Unit and Cancer Research UK Edinburgh Centre, MRC Institute of Genetics & Molecular Medicine, The University of Edinburgh, Western General Hospital, Crewe Road South, Edinburgh EH4 2XU, UK

**Figure:**
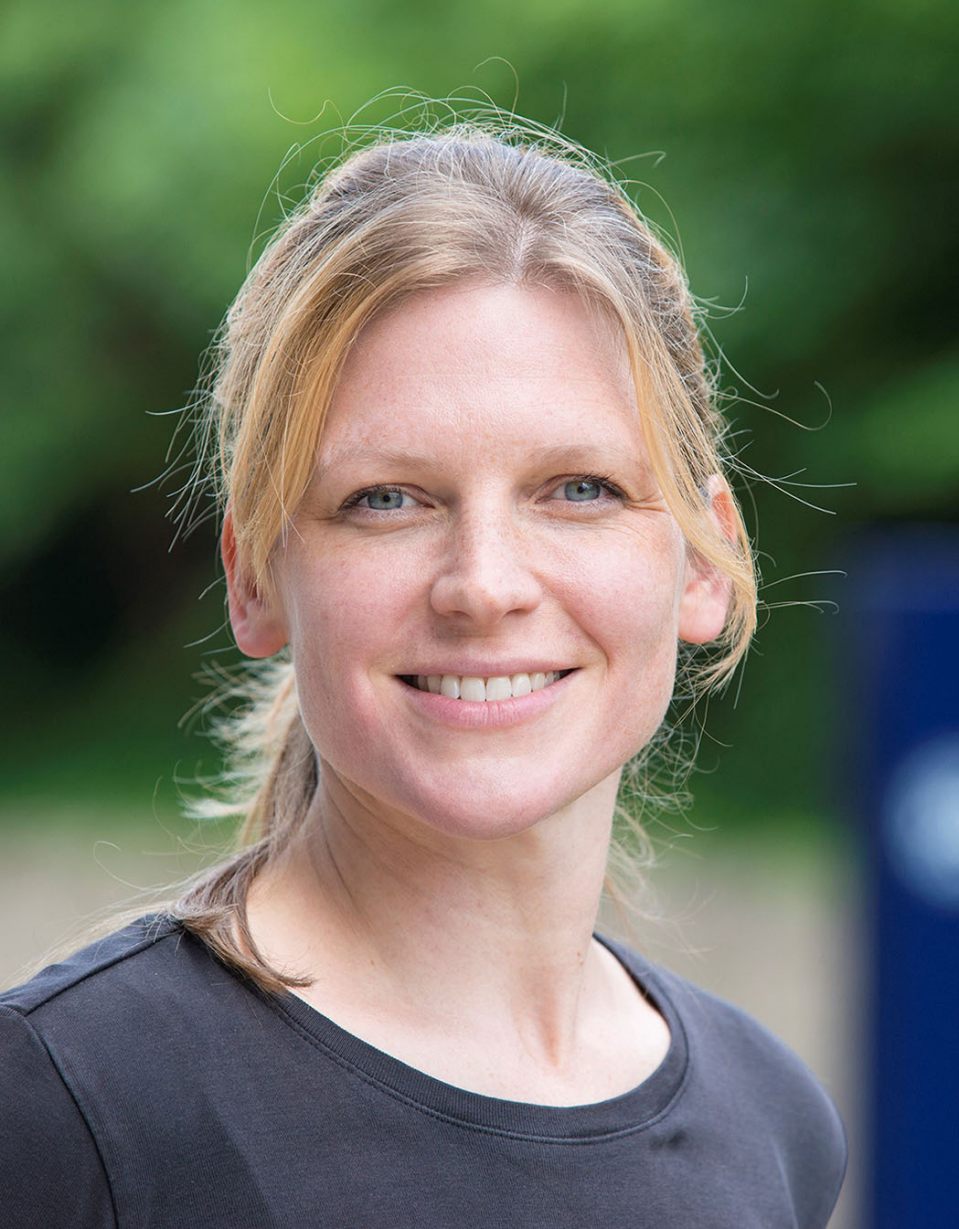
**New DMM Editor-in-Chief Elizabeth Patton.**

This past year, it has become painfully obvious how much we rely on scientific research to protect us from disease and develop the therapies that aid our recovery. We know that the impact of COVID-19 will be with us for some time. We worry about emerging variants bringing additional challenges. But the governmental approval of highly effective vaccines has brought hope to us all. During this pandemic, skilled and determined scientists, and clinical teams across the world rose to the challenge of developing and delivering vaccines as well as therapies for infected individuals at an unprecedented speed. This globally shared experience has brought home how important basic and translational science is for probing disease aetiology and advancing diagnosis and therapy, and how imperative it is that the results of scientific research are freely accessible to anyone and everyone.

These two principles – quality disease research and accessibility – are the twin pillars of Disease Models & Mechanisms (DMM). Founded in 2008, DMM has developed into a leader in publishing fully Open Access research dedicated to disease biology. It is a great honour to be the fourth Editor-in-Chief of DMM, following in the footsteps of Vivian Siegel, Ross Cagan and Monica Justice (Vyas, 2020). Moving forward, I will build on their achievements, which have been instrumental in supporting disease-focused research across model organism communities. As biomedical research has moved into the molecular and genomic eras, there is a high priority for *in vivo* biology to provide insight into disease mechanisms and therapeutic action. Disease models serve as engines for innovative technologies and therapies because they allow us to ask the most pertinent biological questions, predict disease aetiology, progression and resolution, and assess drug responses.

“These two principles – quality disease research and accessibility – are the twin pillars of Disease Models & Mechanisms (DMM).”

I will seek to steer DMM towards pre-clinical research that influences human medical biology and will emphasise the importance of *mechanisms* equally to that of *models*. High-quality research based on broadly defined model organisms has always been central to our DMM vision. However, in consultation with the community, we know we also need to reach scientists with expertise in human cell and tissue models, such as induced pluripotent stem cells, organoids, tissue explants and organotypic cultures, as well as advanced bioengineering and *in vitro* models, such as human organ-chips and bioprinted models. Further, we need to support mechanistic research in infectious disease, including viral, bacterial and parasitic infections. As we engage with these research fields, DMM will encourage new content that addresses the following four thematic challenges that are crucial for advancing the human medical biology field.

“DMM aims to publish high-quality research that teaches us novel aspects of disease biology and will consider any system appropriate that achieves this goal.”

## Mechanisms of disease

Incorporating aspects of the physiological microenvironment and immune system into living systems can reveal novel insights into disease-relevant mechanisms. These aspects are already well represented in DMM by laboratory model organisms but, as mentioned above, DMM also aims to support advances made in models derived from patient materials, such as organoids and tissue-derived models, and in non-traditional systems. Certain disease or injury processes might not lend themselves to mechanistic studies in a model organism, and it may, therefore, be most effective to study the process itself. Infectious disease is one example, with COVID-19 remaining a clear priority but highly prevalent diseases, such as malaria, hepatitis, HIV and tuberculosis, also place a heavy burden on parts of the world already suffering from health disparity. Other conditions include human aging, neurodegenerative disease, and organ injury and dysfunction, where studying proxies for disease processes could complement conventional models. DMM aims to publish high-quality research that teaches us novel aspects of disease biology and will consider any system appropriate that achieves this goal.

For all models, we should consider both their potential for discovery science, and how they can serve as pre-clinical models of disease states and therapeutic outcomes (Patton et al., 2021). To be meaningful, disease models need to be grounded in the appropriate clinical question, capture genetic heterogeneity and incorporate environmental elements, such as diet, and chemical, biological or physical exposures relevant for human biology. The availability of large datasets from patients and model systems provides a wealth of information with which to gauge alignment between models and human disease, and to test new hypotheses.

## Innovative technologies

Some of DMM's most highly read papers report on innovative technologies. Large-scale genomics, single-cell omics, high-throughput screens, gene editing, gene therapy, super-resolution imaging, cryo-electron microscopy and artificial intelligence are just some of the innovative technologies used to interrogate disease models. As the scientific community aspires to understand disease at single-cell resolution, coupling advanced imaging techniques with single-cell RNA and DNA sequencing has revolutionized our understanding of cell states, genetics and lineages linked with disease mechanisms. This is a particularly exciting space for DMM, as technological advances can be leveraged to build more-complex models that may also serve as pre-clinical testing platforms.

## Disease progression through time

Moving beyond snapshots of static disease states, we aim to report on models that capture the patient journey through disease and treatment. This is an area where sophisticated models might reveal novel drug targets and help us to better understand how the body responds to disease resolution and therapy. Our understanding of immune responses to cancer and treatments, drug resistance mechanisms and syndromes that have long-term impact on the quality of life, such as long COVID, is wanting. In addition, deciphering how underlying metabolic syndrome, genetic conditions and mental health disorders affect the body over time and its responses to therapy is crucial for developing effective therapies for millions of affected individuals. Incorporating the time dimension into models is a challenge but will help us achieve a more holistic understanding of disease progression and resolution.

## Therapy

More complex drug-discovery models that enable deep phenotyping of drug actions *in vivo* is a crucial aspect of drug development. The integrated physiology of whole-animal models uniquely informs about on- and off-target effects, therapies for targeted tissues and distal sites, and employs parameters that can help us to better relate targets to drug treatment responses in patients. Hence, whole-animal models are crucial to support innovative therapeutic approaches, including gene therapy, nanotechnology and immunotherapy, as well as their treatment regimens.

“We aspire for DMM to become a natural home for science that pushes the boundaries of disease biology and makes strong links with clinical science.”

Interdisciplinary collaborations between biologists, clinicians, physicists, computational biologists and bioengineers are required to address these four challenges. We aspire for DMM to become a natural home for science that pushes the boundaries of disease biology and makes strong links with clinical science. To expand our expertise, we will recruit new Editors to the team, add expert Associate Editors and complement our Editorial Advisory Board with leaders in disease biology. We are delighted to announce the appointment of new Editors Monkol Lek (Yale School of Medicine, USA) and Rickie Patani (The Francis Crick Institute, London, UK). We will also increase the number of Review and Opinion articles, commissioned from clinical and non-clinical colleagues, and leaders in innovative medicines (all free to read). I am delighted that Elaine Mardis (co-executive director of the Institute for Genomic Medicine at Nationwide Children's Hospital, USA) will serve as our Deputy Editor, helping DMM forge ever-closer links with medical biology and patient communities.

Based on article downloads and citations, we know that DMM readers enjoy in-depth Reviews and At a Glance poster articles. To build on this success, we have added Perspectives – which outline key challenges in a disease field. In this issue, clinician-scientist James Amatruda (Children's Hospital Los Angeles) contributes our first Perspective, writing on the importance of understanding the developmental origins of paediatric cancer to improve outcomes in children, who disproportionately suffer quality-of-life losses after therapy (Amatruda, 2021).

DMM is fortunate to be published by The Company of Biologists, a not-for-profit publishing organisation dedicated to supporting and inspiring the biological community. The distinguished and practising scientists who run the company ensure that its activities are responsive to the community. This includes offering grants, fellowships, and funding and running DMM Workshops and Journal Meetings (Justice et al., 2020; Nicholson and Hmeljak, 2020), launching a new Sustainable Conferencing Initiative, providing a community forum for sharing information on complex technologies (e.g. FocalPlane), supporting the dissemination of science through Open Access publishing and adapting to funder mandates, such as Plan S. We know that much of our research is funded by taxpayers and charities. It is crucial that – whatever their funding body or financial status – DMM authors are able to ensure that their research is freely accessible to all, including researchers, patients and their families, advocates and the wider public.

Beneath the twin pillars of DMM, of quality disease research and freely accessibly science, lies the foundation provided by the in-house editorial team (based in Cambridge, UK), our international research-active scientific Editor team and Editorial Advisory Board, and The Company of Biologists. I am grateful for their commitment to DMM and for sharing our values in scientific publishing. We want to express our thanks to authors for trusting DMM with submissions, and reviewers for their dedication and critical insights (with particular thanks to our 2020 reviewers; see Fig. S1). This past year has shown us what we can do as a global scientific community to combat disease and improve the quality of life, and even save lives. I look forward to working together to build DMM as a leading, freely accessible journal in disease research that improves health and supports our global community.

## Supplementary Material

Supplementary information
