# Supplementary material for: The twin pillars of Disease Models & Mechanisms
Source: Dis Model Mech. 2021 Feb 22;14(2):dmm048951. doi: 10.1242/dmm.048951 (PMC7927655; doi:10.1242/dmm.048951)
Supplement: Supplementary information [file dmm-14-048951-s1.pdf]

Reviewers for Disease Models & Mechanisms 2020

We thank all our reviewers and co-reviewers for their time and expertise.

Trond Aasen, Vall d'Hebron Institut de Recerca (VHIR), Spain

Robert Abramovitch, Michigan State University, USA

Abraham Acevedo Arozena, Hospital Universitario de Canarias, Spain

Kumar Alagramam, University Hospitals Cleveland Medical Center, USA

Matthew Alexander, University of Alabama at Birmingham, USA

Aymon Ali, University of Western Ontario, Canada

Sandra Alves, INSA, National Health Institute Doutor Ricardo Jorge, Portugal

James Amatruda, Children's Hospital Los Angeles, USA

James Amos-Landgraf, University of Missouri, USA

Bogi Andersen, University of California, Irvine, USA

Emma Andersson, Karolinska Institutet, Sweden

Ida Annunziata, St Jude's Children's Research Hospital, USA

Lynda Aoudjehane, Sorbonne Université, France

Nur Arfian, Universitas Gadjah Mada, Indonesia

Hiroyoshi Ariga, Hokkaido University, Japan

Duchon Arnaud, CNRS, France

Timothy Arnett, Division of Biosciences, UCL London, UK

Rene Arvola, The Ohio State University, USA

Atsushi Asakura, Stem Cell Institute, University of Minnesota, USA

Sophie Astrof, Rutgers University, USA

Ruxandra Bachmann-Gagescu, University of Zurich - Institute of Medical Genetics, Switzerland

Michel Bagnat, Duke University, USA

Gurpreet Baht, Duke University School of Medicine, USA

Jeroen Bakkers, Hubrecht institute, The Netherlands

Marica Bakovic, University of Guelph, Canada

Simon Bamforth, Newcastle University, UK

Thomas Baranski, Washington University School of Medicine in St Louis, USA

Ariadna Bargiela, Universitat de Valencia, Spain

Nicholas Barker, A*STAR, IMB, Singapore

Michail Barkoulas, Imperial College London, UK

Peter Bass, Drexel University, USA

Fuller Bazer, Texas A&M University, USA

Catherina Becker, University of Edinburgh, UK

Fariba Behbod, University of Kansas, USA

Hugo Bellen, HHMI - Baylor College of Medicine, USA

Dylan Bergen, University of Bristol, UK

Jason Berman, Dalhousie University, Canada

Olivia Bermingham-McDonogh, University of Washington, USA

Bruce Beutler, University of Texas Southwestern Medical Center, USA

Ujjal Bhawal, Nihon University School of Dentistry at Matsudo, Japan

Bryan Bjork, Midwestern University, USA

Karen Blyth, Beatson Institute, Glasgow, UK

Dirk Bohmann, University of Rochester Medical Center, USA

Johannes Boltze, University of Warwick, UK

Luke Boulter, University of Edinburgh, UK

Marlene Bouvier, University of Illinois Chicago, USA

Melissa Bowerman, University of Oxford, UK

Susan Brain, King's College London, UK

Cristina Branco, Queen's University Belfast, UK

Thomas Brand, Imperial College London, UK

Ralf Braun, Danube Private University, Austria

Jeroen Bremer, University Medical Center Groningen, The Netherlands

Michael Briggs, Newcastle University, UK

Volker Briken, University of Maryland, USA

Julie Brill, The Hospital for Sick Children, Toronto, Canada

Marco Brotto, University of Texas, Arlington, USA

Valerie Brunton, University of Edinburgh, UK

Bryan Bryson, MIT, USA

Rebecca Burdine, Princeton University, USA

Alexa Burger, University of Colorado, USA

Jason Burkhead, University of Alaska, USA

Douglas Burrin, Baylor College of Medicine, USA

Guy Caldwell, The University of Alabama, USA

Kim Caldwell, The University of Alabama, USA

Stephanie Campbell, BC Cancer, Vancouver, Canada

Marina Campione, CNR Institute of Neurosciences, Italy

Thomas Carroll, University of Texas Southwestern Medical Center, USA

James Cassat, Vanderbilt University Medical Center, USA

Michel Cayouette, IRCM, McGill University, Canada

Navdeep Chandel, Northwestern University, USA

Yi-Wen Chen, Children's Research Institute, USA

Kong Chen, University of Pittsburgh, USA

Lili Chen, Icahn School of Medicine at Mount Sinai, USA

Keith Cheng, PennState College of Medicine, USA

Jen-Tsan Chi, Duke University School of Medicine, USA

Judy Cho, Icahn School of Medicine at Mount Sinai, USA

Janice Chou, NICDH, NIH, USA

David Clouthier, University of Colorado, Denver, USA

Seth Coffelt, Glasgow University, UK

Allison Coffin, Washington State University, Vancouver, USA

Bruce Conklin, Gladstone Institutes, USA

Thomas Cooper, Baylor College of Medicine, USA

Jonathan Cooper, Washington University, USA

Antony Cougnoux, NIH, USA

Timothy Cox, University of Missouri, USA

Marija Cvetanovic, University of Minnesota, USA

Esther Dalfo, Universitat Autonoma de Barcelona, Spain

Mark Davenport, King’s College Hospital, London, UK

Clare Davies, University of Birmingham, UK

Alessandra d’Azzo, St. Jude Children’s Research Hospital, USA

Jessica de Greef, Leiden University Medical Center, The Netherlands

José de la Pompa, Centro Nacional de Investigaciones Cardiovasculares Carlos III, Spain

Sofia de Oliveira, Albert Einstein College of Medicine, USA

Hans Degens, Manchester Metropolitan University, UK

Kerry Delaney, University of Victoria, Canada

April DeLaurier, University of South Carolina Aiken, USA

Christos Delidakis, Forth Institute of Molecular Biology and Biotechnology, Greece

Emily Derbyshire, Duke University School of Medicine, USA

Elia Di Schiavi, Institute of Biosciences and BioResources, Napoli, Italy

Albena Dinkova-Kostova, University of Dundee, UK

Luisa DiPietro, University of Illinois Chicago, USA

Chris Doe, University of Oregon, USA

Maria Doitsidou, University of Edinburgh, UK

Patricia Donahoe, Massachusetts General Hospital/Harvard Medical School, USA

Richard Dorsky, University of Utah, USA

James Dowling, Hospital for Sick Children, Toronto, Canada

Gerald Downes, University of Massachusetts Amherst, USA

Nadine Dragin, INSERM Centre de Recherche en Myologie, France

Monica Driscoll, Rutgers, The State University of New Jersey, USA

Carrie Duckworth, The University of Liverpool, UK

Haim Einat, The Academic College of Tel-Aviv Yaffo, Israel

Judith Eisen, University of Oregon, USA

Karin Eisinger, University of Pennsylvania, USA

Florent Elefteriou, Baylor College of Medicine, USA

Phil Elks, University of Sheffield, UK

Nicole Endlich, Institute of Anatomy and Cell Biology, University of Greifswald, Germany

Robert Erickson, University of Arizona, USA

James Eubanks, Toronto Western Research Institute, Canada

Alessandra Eva, IRCCS Istituto Giannina Gaslini, Italy

Todd Evans, Albert Einstein College of Medicine, USA

Louise Evans, University of Minnesota, USA

Sarah Ewald, University of Virginia, USA

Federico Fabrizio, Fondazione IRCCS Casa Sollievo della Sofferenza, Italy

Walid Fakhouri, University of Texas Health Science Center at Houston, USA

Yinshan Fang, Columbia University Medical Centre, USA

Colin Farquharson, Roslin Institute, UK

Donna Fekete, Purdue University, USA

Marie-Anne Félix, Ecole Normale Supérieure, France

Feifei Feng, College of Public Health, Zhengzhou University, China

Hui Feng, Boston University, USA

Olivier Feron, Université Catholique de Louvain, Belgium

Joao Ferreira, Chulalongkorn University, Thailand

Austin Ferro, University of Minnesota, USA

Bonnie Firestein, Rutgers, The State University of New Jersey, USA

Elizabeth Fisher, University College London, UK

Shannon Fisher, Boston University, USA

Mark Fishman, Harvard Stem Cell Institute, USA

Benjamin Fogelgren, University of Hawaii, USA

Wayne Frankel, Columbia University, USA

J. Kimble Frazer, University of Oklahoma Health Sciences Center, USA

James Gagnon, University of Utah, USA

Rene Galindo, UT Southwestern Medical Center, USA

Máximo Galindo, Centro de Investigación Príncipe Felipe, Spain

Rajashekhar Gangaraju, University of Tennessee Health Science Center, USA

Jamie Garcia, Duke University, USA

Vidu Garg, Nationwide Children’s Hospital, USA

Dominique Gauguier, INSERM, France

Daria Gavriouchkina, Okinawa Institute of Science and Technology Graduate University, Japan

Christoph Gerhardt, Heinrich Heine University, Germany

Claire Gibson, University of Nottingham, UK

Bojana Gligorijevic, Temple University, USA

Beatriz Godínez-Chaparro, Universidad Autónoma Metropolitana-Xochimilco, Mexico

Alpaslan Gokcimen, Adnan Menderes University, Turkey

Jeffrey Golden, Harvard University, USA

Pilar Gonzalez-Cabo, University of Valencia, Spain

Misty Good, Washington University School of Medicine in St. Louis, USA

Anthony Graham, King's College London, UK

Susanne Grässel, University of Regensburg, Germany

Ryan Gray, University of Texas at Austin, Dell Medical School, USA

Alex Gregorieff, McGill University, Canada

Eva Gronroos, The Francis Crick Institute, UK

Anya Grosberg, University of California, Irvine, USA

Vandana Gupta, Brigham and Women's Hospital, Harvard Medical School, USA

Melissa Halpern, University of Arizona, USA

Robert Hammer, University of Texas Southwestern Medical Center, USA

Chrissy Hammond, University of Bristol, UK

Jacob Hanna, Weizmann Institute of Science, Israel

Nicholas Hanne, North Carolina State University, USA

Jonathan Hardy, Michigan State University, USA

Stephen Harrap, University of Melbourne, Australia

Bassem Hassan, Flanders Interuniversity, Belgium

Susan Hayflick, Oregon Health & Science University, USA

John Cijiang He, Icahn School of Medicine at Mount Sinai, USA

Denis Headon, Roslin Institute, UK

Joan Heath, Walter and Eliza Hall Institute of Medical Research, Australia

Nicholas Heaton, Duke University School of Medicine, USA

Harry Heimberg, Vrije Universiteit Brussel, Belgium

Alicia Hidalgo, The University of Birmingham, USA

Katsumi Higaki, Tottori University, Japan

Matthew Hind, Imperial College, London, UK

Ellen Hoffman, Yale University School of Medicine, USA

Eric Hoffman, Binghamton University - SUNY, USA

Brigid Hogan, Duke University School of Medicine, USA

Catherine Hogan, Cardiff University, UK

J. Robert Hogg, National Institutes of Health, USA

Paul Hopkins, King's College London, UK

Stacy Horner, Duke University, USA

Yariv Houvras, Weill Cornell Medical College, USA

He Huang, Zhejiang University, China

Simon Hughes, King's College London, UK

Neil Hukriede, University of Pittsburgh, USA

Stacey Huppert, Cincinnati Children's Hospital Medical Center, USA

Robert Hynds, University College London, UK

Michael Ibba, Ohio State University, USA

Akihiro Ikeda, University of Wisconsin-Madison, USA

Matthew Ingalls, University of Rochester, USA

Magnus Ingelman-Sundberg, Karolinska Institutet, Sweden

Andrew Intlekofer, Memorial Sloan Kettering Cance Center, USA

Adonis Ioannides, University of Nicosia Medical School, Cyprus

Zhenya Ivakine, The Hospital for Sick Children, Toronto, USA

Rene Jackstadt, HI-STEM, Germany

Krzysztof Jagla, Université Clermont Auvergne, France

Claudia Jakubzick, Dartmouth Geisel School of Medicine, USA

Morgan James, Rutgers University, USA

Ignacio Jausoro, Y-TEC Tecnologia, Argentina

Lucie Jeannotte, Centre de Recherche du CHU Québec-Université Laval, Canada

Rosalind John, Cardiff University, UK

Jamie Johnston, University of Leeds, UK

Emma Jones, University of Alabama, USA

Monica Justice, The Hospital for Sick Children, Toronto, Canada

Bernadett Kalmer, University College London, UK

Kentaro Kato, Kyorin University School of Medicine, Japan

Kanisha Kavdia, St. Jude Children's Research Hospital, USA

Cristina Keightley, La Trobe University, Australia

Dwi Kemaladewi, Children's Hospital of Pittsburgh, USA

Prashant Khare, AIIMS Bhopal, India

Toshihiro Kitamoto, University of Iowa, USA

Francesca Klinger, Università degli Studi di Roma "Tor Vergata", Italy

Yuliya Klymenko, Indiana University School of Medicine, USA

Ron Korstanje, The Jackson Laboratory, USA

Rashmi Kothary, Ottawa Hospital Research Institute, Canada

Peter Koulen, University of Missouri - Kansas City, USA

Roman Krawetz, University of Calgary, Canada

Markus Kuehn, University of Iowa, USA

Hiroshi Kurosaka, Osaka University Graduate School of Dentistry, Japan

Kristen Kwan, University of Utah, USA

Michael Kyba, University of Minnesota, USA

Lauren Laboissonniere, University of Florida, USA

Mary Jo LaDu, ENH Research Institute, USA

Sarita Lagalwar, Skidmore College, USA

Jennifer Lang, University at Buffalo, USA

David Langenau, Massachusetts General Hospital, USA

Melinda Larsen, University at Albany, State University of New York, USA

Justin Lathia, Cleveland Clinic, USA

Elizabeth Lawrence, University of Bristol, UK

Wei-Dong Le, Institute of Health Science, China

Lance Lee, University of South Dakota, USA

Gabsang Lee, Johns Hopkins University, USA

Frances Lefcort, Montana State University, USA

Louis Lefebvre, University of British Columbia, Canada

Stylianos Lefkopoulos, Max Planck University Freiburg, Germany

Gunter Leuckx, Vrije Universiteit Brussel, Belgium

Yuqing Li, University of Florida, USA

Ellen Lien, University of Southern California, USA

Janghoo Lim, Yale University, USA

Georgie Lines, University College London, UK

Tatiana Lipina, University College London, UK

Lei Liu, Beijing Institute for Brain Disorders, China

Xian-Shuang Liu, Henry Ford Hospital, USA

Zhaoyang Liu, University of Texas at Austin, USA

Emyr Lloyd-Evans, Cardiff University, UK

Jeremy Lotto, University of British Columbia, Canada

Cathleen Lutz, The Jackson Laboratory, USA

Xianjue Ma, Westlake University, China

Cheng-I Jonathan Ma, University of Toronto, Canada

Long Ma, Central South University, China

Calum MacRae, Brigham and Women's Hospital, Harvard Medical School, USA

James Maher, Mayo Clinic, USA

Kelly Miao, Boston University, USA

Giovanni Manfredi, Cornell University, USA

Eduardo Marbán, Cedars-Sinai, USA

Troy Markel, Indiana University School of Medicine, USA

Kurt Marsden, NC State University, USA

Enrique Martín-Blanco, The Institute of Molecular Biology of Barcelona, Spain

Molly Matty, Salk Institute for Biological Studies, USA

Lisa Maves, Seattle Children's Research Institute, USA

Jacqueline Mays, National Institutes of Health, USA

Martin McBride, University of Glasgow, UK

Brian McDermott, Case Western Reserve University, USA

Steven McElroy, University of Iowa, USA

Jodi McGill, Iowa State University, USA

Ian McGough, Francis Crick Institute, UK

Miriam Meisler, University of Michigan, USA

Liliana Mendieta, Benemérita Universidad Autónoma de Puebla, Mexico

Hemmo Meyer, University of Duisburg-Essen, Germany

Yuxuan Miao, University of Chicago, USA

Rachel Miller, McGovern Medical School, USA

Jason Mills, Washington University, USA

Parviz Minoo, Keck School of Medicine USC, USA

Kenji Mishima, Showa University, Japan

Thimios Mitsiadis, Universität Zürich, Switzerland

Masayuki Miura, University of Tokyo, Japan

Bertrand Mollereau, ENS de Lyon, France

Noël Morgan, University of Exeter, UK

Mitsuru Morimoto, RIKEN Center for Biosystems Dynamic Research, Japan

Cara Morin, St. Jude Children's Research Hospital, USA

Jennifer Morton, The Beatson Institute, Glasgow, UK

Christian Mosimann, University of Colorado School of Medicine, USA

Matthew Moulton, Baylor College of Medicine, USA

Victoriano Mulero, Universidad de Murcia, Spain

Erin Mulkearns-Hubert, Lerner Research Institute, Cleveland Clinic, USA

Andreas Müller, Universitat Dresden, Germany

Stephen Murray, The Jackson Laboratory, USA

Kevin Myant, University of Edinburgh, UK

Alexandra Naba, University of Illinois at Chicago, USA

Michio Nagata, University of Tsukuba, Japan

Emi Nagoshi, University of Geneva, Switzerland

Saidas Nair, University of California, San Francisco, USA

Mohandas Narla, New York Blood Centre, USA

Deirdre Nelson, University at Albany, SUNY, USA

Hemanth Ramesh Nelvagal, Washington University St Louis, USA

Brent Neumann, Monash University, Australia

Peter Nghiem, Texas A&M University, USA

Robert Nickells, University of Wisconsin-Madison, USA

Nikolay Ninov, Center for Regenerative Therapies Dresden, Germany

Aleksandra Nita-Lazar, National Institute of Allergy and Infectious Diseases, USA

Dominic Norris, MRC Mammalian Genetics Unit, UK

Brucker Nourse, University of Alabama, USA

Chad Novince, Medical University of South Carolina, USA

Mark O'Driscoll, Sussex Centre for Genome Damage and Stability, UK

Naoki Okamoto, University of California, Riverside, USA

Peter Olinga, University of Groningen, The Netherlands

Peter Oliver, MRC Harwell, UK

Tamas Orban, Research Centre for Natural Sciences, Hungary

Alvaro Ordonez, Johns Hopkins University, USA

Elaine A Ostrander, NHGRI/NIH, USA

Stanislav Ott, Duke-NUS, Singapore

Georg Otto, The UCL Institute of Child Health, UK

Catherine Ovitt, University of Rochester, USA

Vassilis Pachnis, The Francis Crick Institute, UK

Alex Parker, CRCHUM, Universite de Montreal, Canada

Dane Parker, Rutgers University, USA

Hemal Patel, University of California - San Diego, USA

Basant Patel, Indian Institute of Technology, India

Liz Patton, Edinburgh University, UK

Ralf Paus, University of Miami, USA

Louis Penning, Utrecht University, The Netherlands

Rita Perlingeiro, University of Minnesota, USA

Stefano Piccolo, University of Padua, Italy

Stewart Pickering-Brown, The University of Manchester, UK

David Picketts, University of Ottawa, Canada

Adam Pietrobon, University of Ottawa, Canada

Cristina Porcheri, University of Zurich, Switzerland

Lucas Pozzo-Miller, University of Alabama at Birmingham, USA

Michal Pravenec, Czech Academy of Sciences, Czech Republic

Iryna Prots, Friedrich-Alexander-Universität Erlangen-Nürnberg, Germany

Kalyan Prudhvi, Montefiore Medical Center, USA

Sergey Prykhozhij, Dalhousie University, Canada

Bethan Psaila, Radcliffe Department of Medicine, University of Oxford, UK

Alberto Quaglia, Royal Free London NHS Foundation Trust, UK

Jianwen Que, Columbia University, USA

Nuno Raimundo, University Medical Center Goettingen, Germany

Laura Ranum, University of Florida, USA

John Rawls, University of North Carolina School of Medicine, USA

Roger Reeves, Johns Hopkins University School of Medicine, USA

Angeles Ribera, University of Colorado at Anschutz Medical Center, USA

Heather Richbourg, UCSF, USA

Thomas Riemensperger, University of Cologne, Germany

Paul Riley, University of Oxford, UK

Carlo Rinaldi, University of Oxford, UK

Ryan Roberts, Nationwide Children's Hospital, USA

Daniel Rodrigo-Torres, The University of Edinburgh, UK

Russell Rogers, Cedars-Sinai Medical Center, USA

Randall Roper, Indiana University Perdue University Indianapolis, USA

Jason Rosch, St. Jude Children's Research Hospital, USA

Mathias Rosenfeldt, University of Wuerzburg, Germany

Emily Rosowski, Clemson University, USA

Michael Ross, Albert Einstein College of Medicine/Montefiore Medical Center, USA

Daniela Rossi, Universita Di Siena, Italy

Robbert Rottier, Erasmus MC, The Netherlands

Christine Rushlow, NYU College of Arts and Science, USA

Paola Rusmini, University of Milan, Italy

Yukio Saijoh, University of Utah, USA

Jean-Pierre Saint-Jeannet, New York University, USA

Beatriz Salvador, Cardiff University, UK

Nafiseh Sanei, Royan Institute, Islamic Republic of Iran

Susanne Sattler, Imperial College London, UK

Leonor Saude, Instituto de Medicina Molecular, Portugal

Lauren Saunders, University of Washington, USA

Peter Scambler, University College London, UK

Andreas Schedl, Institut de Biologie Valrose, France

Johannes Schlachetzki, University of California, San Diego, USA

Oliver Schmachtenberg, Universidad de Valparaiso, Chile

Heather Schmitt, Duke University, USA

Kris Ann Schultz, Children's Minnesota, USA

Quenten Schwarz, University of South Australia, Australia

Daryl Scott, Baylor College of Medicine, USA

Julie Secombe, Albert Einstein College of Medicine, USA

Sam Senyo, Case Western Reserve University, USA

Praveen Sethupathy, Cornell University, USA

Madhulika Sharma, University of Kansas Medical Center, USA

Cheryl Shoubridge, University of Adelaide, Australia

James Shull, University of Wisconsin-Madison, USA

Guramrit Singh, Ohio State University, USA

Brij Singh, UT Health San Antonio, USA

James Sleigh, University College London, UK

Kelly Smith, University of Melbourne, Australia

Ian Smyth, Monash University, Australia

Michele Solimena, Dresden University of Technology, Germany

Jaewhan Song, Yonsei University, Republic of Korea

Masahiro Sonoshita, Institute for Genetic Medicine, Hokkaido University, Japan

Vincenzo Sorrentino, University of Siena, Italy

Paola Spitalieri, University of Rome Tor Vergata, Italy

Dan Stamer, Duke University, USA

David Stanek, Institute for Molecular Genetics AS CR, Prague, Czech Republic

Amy Stanford, University of Iowa, USA

William Stanford, Ottawa Hospital Research Institute, Canada

Michelle Starz-Gaiano, University of Maryland Baltimore County, USA

Rodney Stewart, University of Utah, USA

Nicola Strenzke, University Medical Center Göttingen, Germany

Henry Sucov, Medical University of South Carolina, USA

Shuying Sun, Johns Hopkins University School of Medicine, USA

David Suter, Ecole Polytechnique Fédérale de Lausanne, Switzerland

Masatoshi Suzuki, University of Wisconsin, USA

Heena Tabassum, Jamia Hamdard, India

Kazue Takahata, Fujimoto Pharmaceutical Corporation, Japan

Pradeep Tanwar, University of Newcastle, Australia

Thomas Tedder, Duke University, USA

Gregory Teicher, University of Massachusetts, USA

Avinash Thakur, University of British Columbia, Canada

Summer Thyme, University of Alabama at Birmingham, USA

David Tobin, Duke University Medical Center, USA

Attila Toth, University of Debrecen, Hungary

David Traver, UCSD, USA

Elizabeth Tucker, Johns Hopkins Hospital, USA

Clare Tweedy, University of Leeds, UK

Jouni Uitto, Sidney Kimmel Medical College at Thomas Jefferson University, USA

Asier Unciti-Broceta, University of Edinburgh, UK

Gokhan Unlu, Rockfeller University, USA

Teresa Valero, University of Edinburgh, UK

Valeria Valsecchi, University of Turin, Italy

Tom Van Agtmael, University of Glasgow, UK

Warren Van Loggerenberg, University of Toronto, Canada

Nuria Vaquero, HI-STEM, Germany

Petr Vodicka, Institute of Animal Physiology and Genetics, Czech Republic

Cindy Voisine, Northeastern Illinois University, USA

Jiaxing Wang, Emory University, USA

Michael Wangler, Baylor College of Medicine, USA

Alastair Watson, University of East Anglia, UK

Sarah Watson, University of Iowa, USA

Conrad Weihl, Washington University, USA

Hendrik Weitkamp, Vanderbilt University, USA

Dominic Wells, Royal Veterinary College, UK

Sabine Werner, Institute of Cell Biology, Switzerland

Robert Wessells, Wayne State University, USA

Grant Wheeler, University of East Anglia, UK

Jonathan Whitchurch, MRC Harwell, UK

Jeffrey Whitsett, Children's Hospital Cincinatti, USA

Miles Wilkinson, University of California San Diego, USA

Michael Wilson, SickKids Research Institute, Canada

Matthew Winter, University of Exeter, UK

Thorsten Wohland, NUS Center for Bioimaging Sciences, Singapore

Selina Wray, University College London, UK

Zhongzhou Yang, Nanjing University, China

Tao Yang, Van Andel Institution, USA

Pamela Yelick, Tufts University, USA

Toshifumi Yokota, University of Alberta, Canada

Y. Eugene Yu, Roswell Park Cancer Institute, USA

Min Yu, University of Southern California, USA

Stephane Zaffran, Aix-Marseille University, France

Chunyue Zhao, Beihang University, China

Jia Qi Cheng Zhang, University of Pittsburgh, USA

Sheng Zhang, University of Texas, USA

Yong Zhang, Chinese Academy of Sciences, China

Xiaoling Zhong, Indiana University, USA

Haining Zhu, University of Kentucky, USA

Anna Zinovyeva, Kansas State University, USA
